# Blocks in the pseudouridimycin pathway unlock hidden metabolites in the *Streptomyces* producer strain

**DOI:** 10.1038/s41598-021-84833-2

**Published:** 2021-03-12

**Authors:** Marianna Iorio, Sahar Davatgarbenam, Stefania Serina, Paolo Criscenzo, Mitja M. Zdouc, Matteo Simone, Sonia I. Maffioli, Richard H. Ebright, Stefano Donadio, Margherita Sosio

**Affiliations:** 1 NAICONS, viale Ortles 22/4, 20139 Milan, Italy; 2 Swammerdam Institute for Life Sciences, University of Amsterdam, Science Park 904, 1098 XH Amsterdam, The Netherlands; 3 Department of Chemistry and Waksman Institute, Rutgers University, Piscataway, NJ 08854 USA

**Keywords:** Industrial microbiology, Metabolomics, Chemical biology, Microbiology

## Abstract

We report a metabolomic analysis of *Streptomyces* sp. ID38640, a soil isolate that produces the bacterial RNA polymerase inhibitor pseudouridimycin. The analysis was performed on the wild type, on three newly constructed and seven previously reported mutant strains disabled in different genes required for pseudouridimycin biosynthesis. The results indicate that *Streptomyces* sp. ID38640 is able to produce, in addition to lydicamycins and deferroxiamines, as previously reported, also the lassopeptide ulleungdin, the non-ribosomal peptide antipain and the osmoprotectant ectoine. The corresponding biosynthetic gene clusters were readily identified in the strain genome. We also detected the known compound pyridindolol, for which we propose a previously unreported biosynthetic gene cluster, as well as three families of unknown metabolites. Remarkably, the levels of most metabolites varied strongly in the different mutant strains, an observation that enabled detection of metabolites unnoticed in the wild type. Systematic investigation of the accumulated metabolites in the ten different *pum* mutants identified shed further light on pseudouridimycin biosynthesis. We also show that several *Streptomyces* strains, able to produce pseudouridimycin, have distinct genetic relationship and metabolic profile with ID38640.

## Introduction

Despite multiple decades of intensive screening, newly identified microbial natural products still represent the best source of life-saving drugs, such as antibacterial and antitumor compounds^1^. These “specialized metabolites”, as microbial natural products are often called, have multiple biological activities that have become useful for humans and usually also have roles in microbial competition, microbial predation, in nutrient uptake and in cell–cell communication^2^. Specialized metabolites produced by soil-dwelling bacteria are especially noteworthy in this regard.

Several microbial genera belonging to different orders within the phylum Actinobacteria, commonly called actinomycetes, hold the genetic information for the synthesis of numerous secondary metabolites, devoting up to 10% of their genomes to this^3,4^. The availability of multiple genome sequences and a variety of analysis tools such as antiSMASH, PRISM and BIG-SCAPE/CORASON allow the rapid identification of biosynthetic gene clusters (BGCs) in bacterial genomes^3–5^. While genomic analyses are progressing fast, the majority of BGCs remain experimentally uncharacterized and yet to be associated to the cognate specialized metabolites. Different methods have been explored to define and harness this biosynthetic potential, including cultivating strains in the presence of elicitors or stress substances^6,7^, modifying BGC promoters^8^, manipulating BGC specific regulators^9^ and deploying decoys of BGC repressors^10^. In all these approaches, the detection of secondary metabolites heavily relies on liquid chromatography (LC) coupled to mass spectrometry (MS). Significant advances are being made in metabolomics, including the development of new tools for organizing and analyzing MS data and databases, making "omics" approaches very useful for prioritizing strains or molecules for further investigations^11–13^.

*Streptomyces* sp. ID38640, isolated from an Italian soil sample, is the producer of pseudouridimycin (PUM), the first selective nucleoside analog inhibitor of bacterial RNA polymerase, endowed with promising activity against Gram-positive and Gram-negative bacteria^14^. PUM is part of the *C*-nucleosides antibiotic family, which also includes formycin, malayamycin and ezomycin^15,16^. In previous work, we analyzed the PUM biosynthetic pathways through knockouts of several *pum* genes present within the PUM BGC, providing the first elucidation of a biosynthetic pathway for a *C*-nucleoside antibiotic^17^. This work has also showed that the pseudouridine synthase PumJ, the key biosynthetic enzyme in the PUM pathway, is present in diverse, taxonomically unrelated microorganisms, suggesting a widespread distribution of yet-to-be-discovered additional *C*-nucleoside antibiotics^17^.

Blocking PUM biosynthesis in the producer strain *Streptomyces* sp. ID38640 led to altered production of the siderophore desferroxiamine and of the polyketide lydicamycin, two unrelated specialized metabolites^17^. Here, we extend these findings by a systematic evaluation of MS profiles in available and *ad-hoc-*generated *pum* mutants, correlating metabolites with corresponding BGCs. Overall, we were able to define seven metabolite-BGC pairs, including proposing an uncovered BGC for a known metabolite. We also show that PUM can be easily detected in diverse *Streptomyces* strains harboring the *pum* BGC. Finally, our analyses provide additional insights into the PUM biosynthetic pathway.

## Results and discussion

### Metabolomic analysis of pum mutants

The functions of seven genes in the PUM gene cluster was previously assigned through bioinformatic analysis and the intermediates detected in knockout mutants [17], as summarized in Table 1. Briefly, the pseudouridine synthase PumJ catalyzes *N*- to *C*-nucleoside isomerization to yield pseudouridine or a derivative thereof, which is then converted into 5′-amino-5′-deoxy pseudourydine (APU) by the action of the oxidoreductase PumI and the aminotransferase PumG. In a converging step, guanidinoacetate (GAA) is produced by the amidino transferase PumN. Next, the amide ligases PumK and PumM add sequentially glutamine and GAA to APU, while PumE catalyzes *N*-hydroxylation (Table 1).

**Table 1:** *pum* mutants and accumulated intermediates. Concentrations (µM) of all intermediates except GAA were measured against a PUM standard. GAA concentrations expressed as ratios to the accumulation in the WT strain. The highest production of each metabolite is in bold type. See text for abbreviations. *ND *not detected.

Mutant	Role	PUM	Deoxy-PUM	OH-Gln-APU	Gln-APU	APU	PU	GAA	References
WT		**200**	**30**	**23**	ND	ND	ND	1	
*ΔpumE*	*N*-hydroxylase	ND	24	ND	66	13	0	1.3	^17^
*ΔpumF*	Regulator	ND	ND	ND	ND	146	58	**2.8**	This work
*ΔpumG*	PUA aminotransferase	ND	ND	ND	ND	ND	51	0.3	^17^
*ΔpumH*	Adenylate kinase	ND	ND	ND	ND	ND	ND	2.5	This work
*ΔpumI*	PU 5′ oxidase	ND	ND	ND	ND	ND	ND	0.8	^17^
*ΔpumJ*	Pseudouridine synthase	20	ND	ND	ND	ND	ND	1.5	^17^
*ΔpumK*	Gln-APU carboxylate-amine ligase	ND	ND	ND	ND	**154**	**138**	1.8	^17^
*ΔpumL*	EXPORT	16	13	4	21	9	ND	1	This work
*ΔpumM*	GAA and Gln-APU amide ligase	ND	ND	8	**136**	48	ND	2.5	^17^
*ΔpumN*	GAA formation	ND	ND	10	76	51	ND	ND	^17^

**Figure 1:**
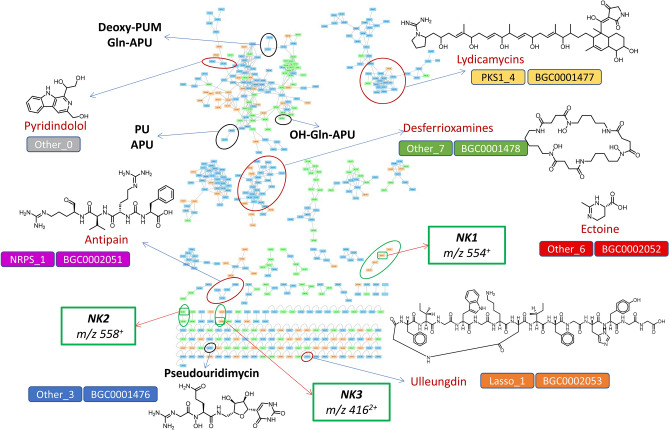
Molecular network of samples from *Streptomyces* sp. ID38640 and ten knockout *pum* mutants. The analysis includes two cultures from each strain and two samples per culture. Node colors give the contributing medium: orange for M8, green for PumP1, light blue for nodes observed in both media. Black circles indicate PUM-related nodes, red circles indicate nodes corresponding to known compounds, green circles show unknown metabolites (NK). The associated BGCs, as listed in Table 2, are shown next to each metabolite.

In order to analyze globally the metabolome of *pum* knockout mutants, we cultivated the wild type producer strain, *Streptomyces* sp. ID38640 (WT) along with the seven previously reported knockout mutant strains (*ΔpumE, ΔpumG, ΔpumI, ΔpumJ, ΔpumK, ΔpumM,* and *ΔpumN*) and three newly constructed ones (*ΔpumF, ΔpumH *and *ΔpumL*; see below), all unable to produce PUM but accumulating different intermediates (Table 1). Each strain was cultivated in two media and we analyzed the metabolite distribution by LC–MS at 24-h intervals over four days. Samples were analyzed after solvent extraction of the whole culture (FE samples; usually hydrophobic metabolites) and also by direct analysis of the cleared broth (SN samples; both hydrophobic and hydrophilic metabolites). The LC–MS/MS data from 44 samples, derived from 11 strains cultivated in two different media with two samples per culture, were subjected to the Global Natural Products Social (GNPS) molecular networking analysis^11^ and visualized using Cytoscape^18^. This analysis clusters spectra having identical MS^2^ patterns, forming nodes (rectangles in Fig. 1) and connects nodes having fragmentation patterns sharing at least 4 fragments (lines in Fig. 1). This results in the formation of networks, representing families of potentially related metabolites. Analysis with Cytoscape provides an intuitive visualization of metabolite distribution according to strain, medium or sample.

The molecular network of Fig. 1, which was derived from 72-h samples, consists of 475 features, including media components, 369 of which are assigned to 36 molecular families, and 106 of which are singletons. Similar results were observed when analyzing samples at different time points, but the 72-h samples the highest metabolite richness. The two tested media afforded equivalent numbers of signals, each showing about 20% unique signals. Each *pum* knockout mutant strain contributed with 200–300 signals each, with *ΔpumK* and *ΔpumH* being at the upper and the lower end, respectively. Similarly, ~ 50% of signals were shared between the FE and SN samples, with ~ 25% unique signals contributed by each sample.

### Correlating metabolites with biosynthetic gene clusters

From a draft genome of *Streptomyces* sp. ID38640, the genome mining tool antiSMASH 5.0^3^ revealed 27 distinct regions harboring BGCs and a predicted metabolic diversity including five non-ribosomal peptides, five polyketides, seven ribosomally synthesized post translationally modified peptides, four terpenes, as well as at least nine regions classified as "other" (Table 2). Overall, 11 BGCs find a match with related BGCs in the MIBiG database.

**Table 2:**
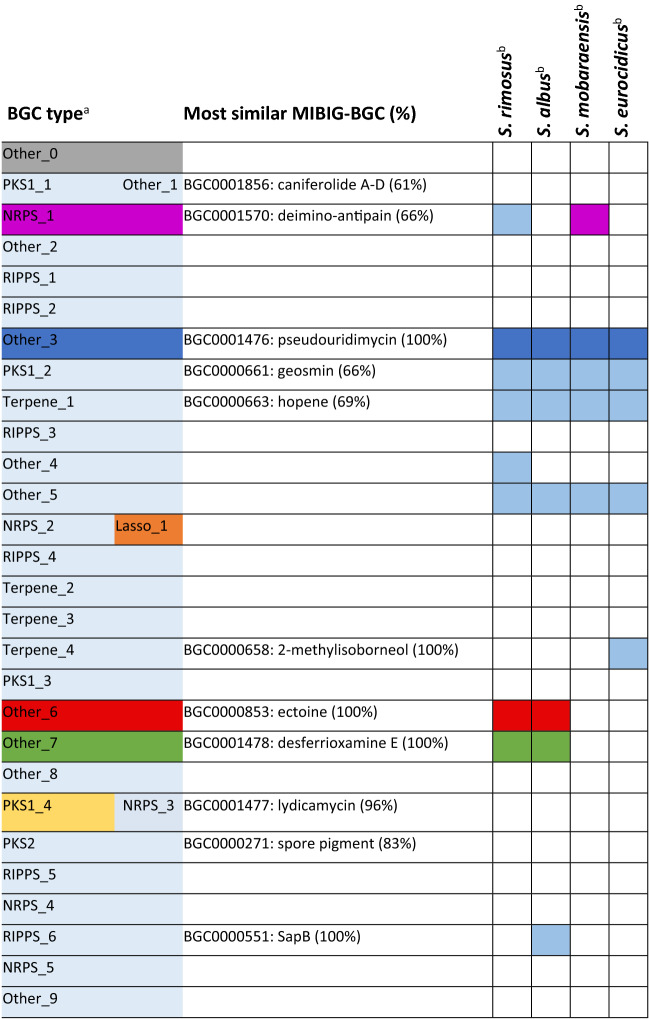
BGCs identified in ID38640 and presence of these BGCs in the four PUM producers.

^a^Regions identified by antiSMASH. Note that "Other0" was identified as explained in text. BGCs with detected metabolites are color-coded as in Fig. 1

^b^Presence of conserved BGCs in the genomes of the four analyzed PUM producers’. BGCs with detected metabolites are color-coded as in Fig. 1, the others are in light blue.

Consistent with our previous work^17^, we detected molecular families corresponding to the lydicamycins and desferroxiamines in all tested samples from each fermentation medium (Fig. 1, Table 3). Particularly, high levels of lydicamycins and desferrioxamines were detected in the *ΔpumI* and *ΔpumN* mutants (Table 3; Supplementary Fig. 1).

**Table 3:** Relative amounts of the identified metabolites in the different *pum* mutants. Amounts are expressed as ratios to those observed in the WT strain in the same medium. Note that for NK1 through NK3, which are not detected in the WT, their presence is indicated with an "X". The highest relative amounts of metabolites are in bold type.

Mutant	Major accumulated PUM metabolite(s)	Desferrioxamine	Pyridindolol	Ectoine	Antipain	Ulleungdin	Lydicamycin	NK1	NK2	NK3
WT	PUM	1.0	1.0	1.0 (traces)	**1.0**	1.0 (traces)	1.0	ND	ND	ND
*ΔpumE*	Gln-APU	2.0	0.8	9.0	ND	**20.0**	0.1	X	ND	ND
*ΔpumF*	APU	1.8	0.9	5.4	0.6	1.0	1.8	ND	ND	ND
*ΔpumG*	PU	1.3	ND	5.4	0.8	1.0	0.1	**X**	**X**	ND
*ΔpumH*	GAA	2.9	ND	7.4	ND	7.0	0.1	ND	ND	ND
*ΔpumI*	GAA	1.7	1.2	1.0	ND	3.0	**3.2**	ND	ND	ND
*ΔpumJ*	GAA	2.5	ND	9.6	0.9	1.0	0.4	ND	ND	ND
*ΔpumK*	PU and APU	2.9	1.4	**10.6**	0.1	5.0	1.2	ND	ND	ND
*ΔpumL*	Gln-APU	2.1	ND	2.0	0.6	1.0	0.1	ND	X	**X**
*ΔpumM*	Gln-APU	2.7	0.6	4.4	0.9	1.0	0.5	X	ND	ND
*ΔpumN*	Gln-APU	**3.3**	**2.5**	3.0	0.4	2.0	1.2	X	ND	ND
Best medium		M8	PumP1	PumP1	Both	M8	Both	M8	PumP1	M8

We also detected a molecular family with a node at *m/z* 303 [M + 2H]^2+^ (Fig. 1), the HR-MS fragmentation pattern and UV spectrum of which (Supplementary Fig. 2) match those of the ureylene-containing oligopeptide antipain, a family identified used an authentic standard in previous work^19^. This family of compounds is produced by non-ribosomal peptide synthetases in numerous bacteria and functions as a protease inhibitor^20^. Consistently, we located a corresponding BGC in the ID38640 genome, that exhibits 61–78% gene sequence identity with antipain BGCs in the MIBIG database^21^ (Table 2). The antipain molecular family is found in both the WT strain and in most *pum* mutants, in both media, with highest amounts detected in the WT strain (Table 3; Supplementary Fig. 1).

A self-loop feature (Fig. 1) was detected with an exact mass corresponding to ulleungdin, a recently reported lassopeptide from *Streptomyces* sp. KCB13F003^22^ (found *m/z* 796.8835 [M + 2H]^2+^; calculated *m/z* 796.8859 [M + 2H]^2+^). The identification was consistent with the MS fragmentation pattern (Supplementary Fig. 3) and with a lassopeptide BGC (Table 2; Supplementary Fig. 3) encoding a predicted core peptide identical to ulleungdin. Ulleungdin was detected in all tested strains, in both fermentation media, with the WT strain showing trace levels (Table 3; Supplementary Fig. 1).

The molecular networking analysis of Fig. 1 was carried out using a cosine score above 0.7. This value was also used to form MS clusters. This filter excluded ectoine, a methyl, tetrahydropyrimidinecarboxylic acid that protects many bacterial species from osmotic stress, since this metabolite shows very poor fragmentation. Nonetheless, we were able to identify a peak, eluting at 1.1 min, consistent with ectoine hydrophilicity, having a matching exact mass (found *m/z* 143.0813 [M + H]^+^, calculated *m/z* 143.0815 [M + H]^+^; Supplementary Fig. 4). Consistently, we located a BGC in the ID38640 genome corresponding to the ectoine BGC (Table 2). Ectoine is detected in all tested strains (Table 3; Supplementary Fig. 1).

Additionally, the molecular network of Fig. 1 highlighted three other molecular families corresponding to unknown molecules. Four different *pum* knockout mutant strains, when grown in M8 medium, produced four related metabolites with *m/z* 536 [M + H]^+^, *m/z* 554 [M + H]^+^, *m/z* 555 [M + H]^+^ and *m/z* 642 [M + H]^+^ (“NK1” in Table 3; Supplementary Fig. 5). Two additional signals, *m/z* 558 [M + H]^+^ and *m/z* 530 [M + H]^+^, were observed in samples from the *ΔpumG* and *ΔpumL* strains, when grown in PumP1 medium (“NK2” in Fig. 1; Table 3; Supplementary Fig. 5). Moreover, two signals corresponding to doubly charged masses *m/z* 416 [M + 2H]^2+^ and *m/z* 423 [M + 2H]^2+^ were observed in samples from *ΔpumL* in M8 medium (“NK3”in Fig. 1; Table 3; Supplementary Fig. 5). No matches of these signals were found in the Dictionary of Natural Products or in the Natural Product Atlas^23,24^, and our internal set of 5200 *Streptomyces* molecular fingerprints indicated that NK1, NK2 and NK3 are rare occurrences. Thus, these molecules may represent novel metabolites worthy of further investigations.

We also observed a molecular family with *m/z* 259 [M + H]^+^ and *m/z* 421 [M + H]^+^, with exact masses of *m/z* 259.1089 [M + H]^+^ and 421.1615 [M + H]^+^, respectively (Supplementary Fig. 6). The associated LC peaks show a UV–Vis spectrum with absorption maxima at 254, 304 and 370 nm. These properties match those of pyridindolol and pyridindolol glucoside, produced by *Streptomyces alboverticillatus* and *Streptomyces parvulus*, respectively^25,26^. The MS2 fragmentations are consistent with this annotation (Supplementary Fig. 6). These metabolites are detected in almost all strains, with the *ΔpumN* mutant showing highest levels (Table 3; Supplementary Fig. 1).

**Figure 2:**
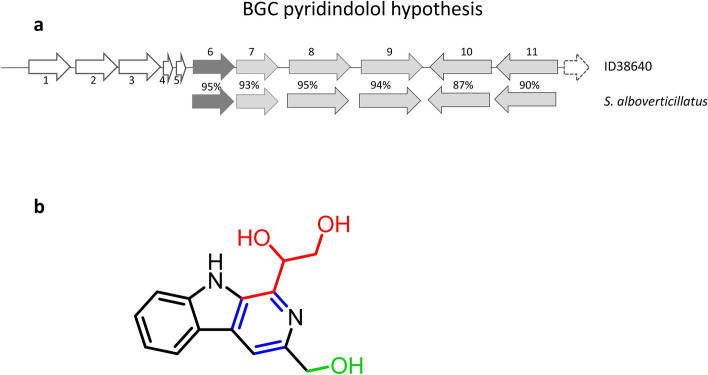
Putative pyridindolol BGC and hypothetical roles in metabolite formation. (**a**) Identified region containing the PSase homolog (dark grey) from the ID38640 genome (top) and syntenic region found in the pyridindolol producer *S. alboverticillatus* (bottom). The percent identities between orthologs are shown, while deduced functions are reported in Table 4. (**b**) Pyridindolol structure, highlighting the likely building blocks tryptophan (black) and glyceraldehyde (red), and the modifications necessary, aromatization (blue) and reduction (green), for the final structure.

Pyridindolol biosynthesis has not been studied previously and no BGC possibly linked to this metabolite could be found in the antiSMASH output. However, it has been reported that the β-carboline moiety present in pyridindolol is formed by a “Pictet-Spenglerase” (PSase), an enzyme that joins an amino group with an aldehyde^27^. The enzyme StnK2 has been shown to function as a PSase in streptonigrin biosynthesis in streptomycetes^28^. Accordingly, we searched the ID38640 genome for StnK2 homologs and identified QIK04791.1 as having 49% sequence identity with StnK2 (Fig. 2; Table 4). The QIK04791.1-encoding region also specifies for a FAD-binding oxidoreductase, a long-chain fatty acid-CoA ligase, an aldehyde dehydrogenase, a histidine phosphatase and a F420-dependent oxidoreductase (Table 4). While most of these sequences have no paralogs in the streptonigrin BGC, we found that a syntenic region with over 90% protein-to-protein identity (Fig. 2; Table 4) is present in the genome of the pyridindolol producer *Streptomyces alboverticillatus* (MUFU00000000; Fig. 2; Table 4). Based on our observations, we hypothesize that pyridindolol formation entails condensation of tryptophan with a C-3 unit, possibly glyceraldehyde(phosphate) by the PSase; aromatization of the newly formed ring by the FAD-binding- and/or the F420-dependent oxidoreductase; and reduction of the carboxyl group by the aldehyde dehydrogenase (Fig. 2). The order in which the hypothesized reactions occur awaits further analysis, as does the possible role of the conserved long-chain fatty acid-CoA ligase and histidine phosphatase present in the conserved segment. This BGC, which lies at one end of the genome sequence in *Streptomyces* sp. ID38640, has been added to Table2 and designated "Other0".

**Table 4:** The proposed pyridindolol BGC.

CDS	Size and protein ID	Protein family	Best MIBIG match, % identity (BGC)	Homolog^a^ [strain, accession no., % identity]
1	326 aa QIK04786.1	Regulator		AraC family transcriptional regulator [*Streptomyces lydicus*, WP_127154781.1, 98%]
2	406 aa QIK04787.1	Flavin reductase		FAD-binding oxidoreductase [*S. lydicus*, WP_127154780.1 97%]
3	574 aa QIK04788.1	Flavin reductase		Flavin reductase [*Streptomyces libani*, WP_159483585.1, 97%]
4	94 aa QIK04789.1	Hypothetical		Lantibiotic dehydratase C-term region [S. rimosus, WP_125052965.1, 73%]
5	90 aa QIK04790.1	Hypothetical		Hypothetical prot [*S. lydicus*, AZS70204, 83%]
6	322 aa QIK04791.1	Pictet-Spenglerase	StnK2, 49% (BGC0001783)	Hypothetical protein [*S. alboverticillatus* WP_086571431.1, 95%]
7	370 aa QIK04792	FAD-binding oxidoreductase	StnP2, 39% (BGC0001783)	FAD-binding oxidoreductase [*S. alboverticillatus*, WP_086571429, 93%]
8	352 aa QIK10964.1	Long-chain fatty acid–CoA ligase		AMP-binding protein [*S. alboverticillatus,* WP_086571427.1, 95%]
9	453 aa QIK04793.1	Aldehyde dehydrogenase family protein		Aldehyde dehydrogenase family protein [*S. alboverticillatus*, WP_086571425.1, 94%]
10	218 aa QIK10965.1	Histidine phosphatase family protein		Histidine phosphatase family protein [*S. alboverticillatus*, WP_086571443.1, 87%%]
11	293 aa QIK04794.1	LLM class F420-dependent oxidoreductase		LLM class F420-dependent oxidoreductase [*S. alboverticillatus*, WP_086571441.1, 90%]
12		IS5/IS1182 family transposase		

^a^ Matches to the syntenic *S. alboverticillatus* region shown in Fig. 2 are in red.

Overall, our work led to the identification of seven metabolite-BGC pairs (Table 2). This leaves 21 BGCs orphan of their product, including those for geosmin and methylisoborneol, volatile metabolites unlikely to be detected under our conditions, and hopene, unlikely to be present in our samples because of its lipophilicity. Thus, it remains to be determined whether these three metabolites are actually produced by *Streptomyces* sp. ID38640. Overall, 18 BGCs await matching metabolites and 3 identified metabolites are missing a matching BGC.

Notably, this work has demonstrated that changes in a small region of the genome facilitate the detection of additional metabolites. It has been previously reported that blocking biosynthesis of a specialized metabolite can facilitate detection of novel chemistry^29^. However, we are not aware of studies showing that different blocks in a single pathway can significantly alter the metabolite levels of biosynthetically unrelated metabolites. Accumulation of a particular PUM intermediate does not appear the reason for altering the metabolic profiles: for example, ectoine levels are tenfold enhanced in the *ΔpumE*, *ΔpumJ* and *ΔpumK* mutants that accumulate different PUM intermediates (Table 1). At the same time, not all mutants accumulating the same PUM intermediate show similar increases in ectoine levels. Thus, the observed modulation of specialized metabolite levels could result from the introduction of the apramycin resistance cassette with its strong promoter, from increased availability of precursors and/or from altered transcription by PUM. The mechanism(s) leading to altered metabolic profiles are currently unknown and further work will be necessary to establish whether this phenomenon is an oddity of the PUM pathway. Nonetheless, the generation of distinct mutants from a single BGC might be useful not only for elucidating the corresponding biosynthetic pathway (see below), but also for detecting chemistry hidden in wild type strain.

### Additional insights into PUM biosynthesis

The functions of most of the *pum* genes and the general pathway of PUM biosynthesis have been defined from our previous in vivo experiments and bioinformatic analyses^17^, as summarized in Table 1. In this work, we generated knockout mutants in three additional *pum* genes: *pumF*, *pumH* and *pumL* (Table 1). PumF shows 42–45% identity to SsaA and its orthologues NpsM and PacA, which are regulators of BGC for the structurally related uridyl-peptide antibiotics sansanmycins, napsamycin and pacidamycin, respectively^30^. PumH, annotated as an adenylate kinase, shares 42% identity with PolQ2 and MalE from the polyoxin and malayamycin biosynthetic pathways, respectively^31^. PumL shares 65% identity with a NocH-like protein belonging to the major facilitator superfamily.

Replacement of *pumF* with the apramycin resistance gene abolished PUM production and led to the accumulation of pseudouridine (PU), amino pseudouridine (APU) and guanidine acetate (GAA) (Table 1; Supplementary Fig. 7). These results indicate that PumF is a positive regulator of PUM production that controls the conversion of APU into Gln-APU. The *ΔpumL* knockout mutant resulted in very low yields of PUM and accumulation of several PUM intermediates, consistent with a role of PumL in exporting the final pathway product (Table 1; Supplementary Fig. 7).

The phenotype of the *ΔpumH* mutant was more complex: it accumulated no PUM-related metabolite except for GAA (Table 1; Supplementary Fig. 7); and, unlike the *ΔpumJ* strain^17^, PUM production could not be rescued by adding PU to the production medium. Thus, the *ΔpumH* phenotype was identical to that of the previously reported *ΔpumI* mutant^17^, which likewise accumulated no intermediate except for GAA and could not convert PU into PUM. The simplest interpretation of these results is that inactivation of *pumH* or *pumI* through insertion of an apramycin resistance cassette has a polar effect on expression of downstream genes in the same transcriptional unit. Consistent with this interpretation, *pumH* and *pumI* overlap by 20 bp, and *pumI* and *pumJ* by 4-bp, suggesting they belong to a single transcriptional unit. Insertion of the apramycin resistance cassette into the kinase-encoding *pumH* would thus disable also the oxidoreductase PumI and the pseudouridine synthase PumJ, while insertion into *pumI* would leave only *pumH* intact. Recently, Draelos et al. have demonstrated 2′-phosphorylation of early intermediates in the nikkomycin and polyoxin pathways by enzymes belonging to the same family as PumH^32^, corroborating the hypothesis that intermediate phosphorylation also occurs during PUM biosynthesis.

We also assessed PUM and PUM precursors accumulated in the WT strain and in the ten *pum* knockout mutants in the molecular network. PUM (*m/z* 487 [M + H]^+^) appears as a single loop detected only in the WT strain and, to a lesser extent, in the *ΔpumJ* and *ΔpumL* mutants, irrespective of the cultivation medium (Fig. 1 and Table 1). We also detected a molecular family including PU, *m/z* 244 [M + H]^+^, and APU, *m/z* 245 [M + H]^+^. PU accumulates only in the *ΔpumF*, *ΔpumG* and *ΔpumK* mutants, while APU accumulates mainly in the *ΔpumF* and *ΔpumK* mutants, with highest levels of both metabolites observed in *ΔpumK* (Table 1). The late PUM intermediates Gln-APU (*m/z* 372 [M + H]^+^) and deoxy-PUM (*m/z* 471 [M + H]^+^) cluster together (Fig. 1). As expected, Gln-APU is found in samples from the *ΔpumE*, *ΔpumL, ΔpumN* and *ΔpumM* strains, with the latter strain accumulating the highest level. Deoxy-PUM was detected in samples from the WT, *ΔpumE* and *ΔpumL* strains, with highest level present in WT, in agreement with previous results (Fig. 1, Table 1). In a different portion of the same network we observed a signal at *m/z* 388 [M + H]^+^) consistent with *N*-hydroxy-Gln-APU (OH-Gln-APU), which corresponded to a hydrophilic peak with the pseudouridine-characteristic UV maximum at 263 nm. This species, which had not been noted in previous work because of its low abundance (Table 1), was detected in samples from the WT and, in lower amounts, from the *ΔpumL*, *ΔpumM* and *ΔpumN* strains. The levels of the PU-containing intermediates sharing the same chromophore were quantified as reported in Table 1.

**Figure 3:**
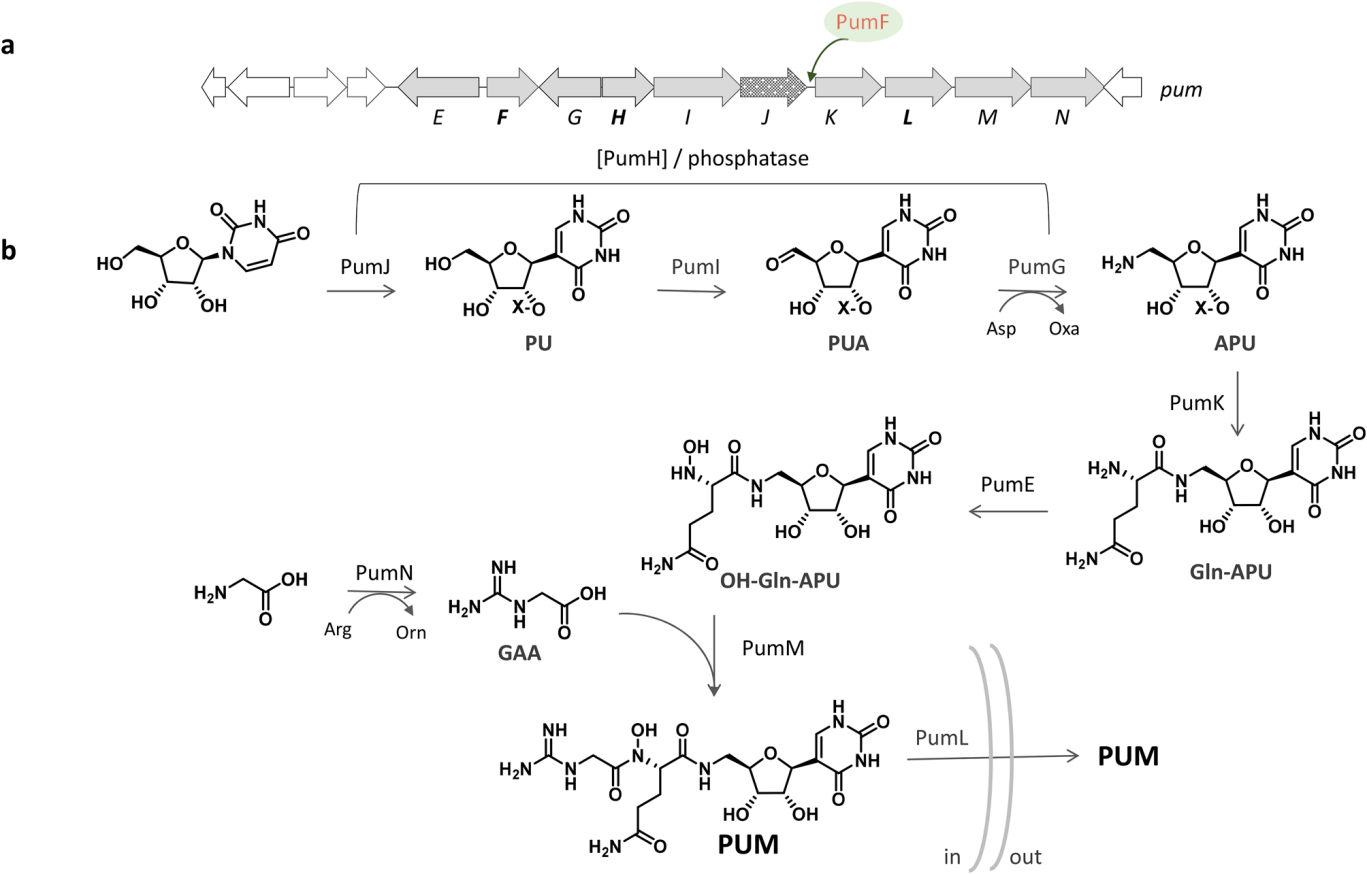
Revised biosynthetic pathway for pseudouridimycin. (**a**) PUM BGC, with established role for PumF. (**b**) Proposed pathway. Enzymes and intermediates reported within brackets have not been experimentally determined. X = H or PO_3_H_2_. Phosphorylation can be introduced by PumH after PU formation and before Gln addition. See text for abbreviations.

The results presented here enable us to confirm and extend the previously proposed biosynthetic pathway for PUM^17^ (Fig. 3). During the early biosynthetic steps, the substrate for the kinase PumH may be either uridine, PU or PU aldehyde (Fig. 3), with PumJ, PumI and PumG acting sequentially for *C-*isomerization, alcohol oxidation and amine formation, respectively. By analogy with the nikkomycin/polyoxin pathways^32^, phosphorylation is likely to occur at the 2′ position, as shown in Fig. 3. Subsequently, the phosphate group is removed by PumD or by a housekeeping phosphatase. A key step in the pathway appears to be the conversion of APU into Gln-APU by PumK, a conversion controlled by the regulator PumF. The detection of OH-Gln-APU suggests that *N*-hydroxylation by PumE precedes addition of GAA by PumM, consistent with the well-known facilitated hydroxylation of amines with respect to amides^33^. In the absence of PumE, PumM uses Gln-APU as substrate, leading to deoxyPUM as a shunt metabolite. While in the absence of PumM, Gln-APU preferentially accumulates, suggesting that either conversion of Gln-APU into its hydroxyderivative is inefficient or that expression of *pumE* is altered in this context. Finally, PumL appears to be a transporter for PUM.

### PUM production by other streptomycetes

In previous work, we identified *pumJ*-related sequences linked to putative BGCs in numerous microbial genomes and we predicted these BGCs specify biosynthesis of PUM or closely related metabolites^17^. Production of PUM has been previously demonstrated only for *Streptomyces* spp. ID38640 and ID38673, from the NAICONS collection^14^, and for *Streptomyces albus* DSM 40763^34,35^. To investigate whether the strains harboring a PUM BGC did produce PUM and other metabolites shared with *Streptomyces* sp. ID38640, we investigated four *Streptomyces* strains: *S. rimosus* ATCC 10970, producer of oxytetracycline; *S. mobaraensis* DSM 40847, producer of the NADH reductase inhibitor piericidin; *S. eurocidicus* ATCC 27428, producer of the antifungal polyene eurocidin; and *S. flocculus* DSM 40313, producer of the aminoquinone antibiotic streptonigrin. [*S. flocculus* has been recently reclassified^36^ and will be referred as *S. albus* DSM 40313 hereafter.] These compounds have been known for several decades and the producer strains have been investigated by several laboratories, but PUM production has to our knowledge not been observed.

**Figure 4:**
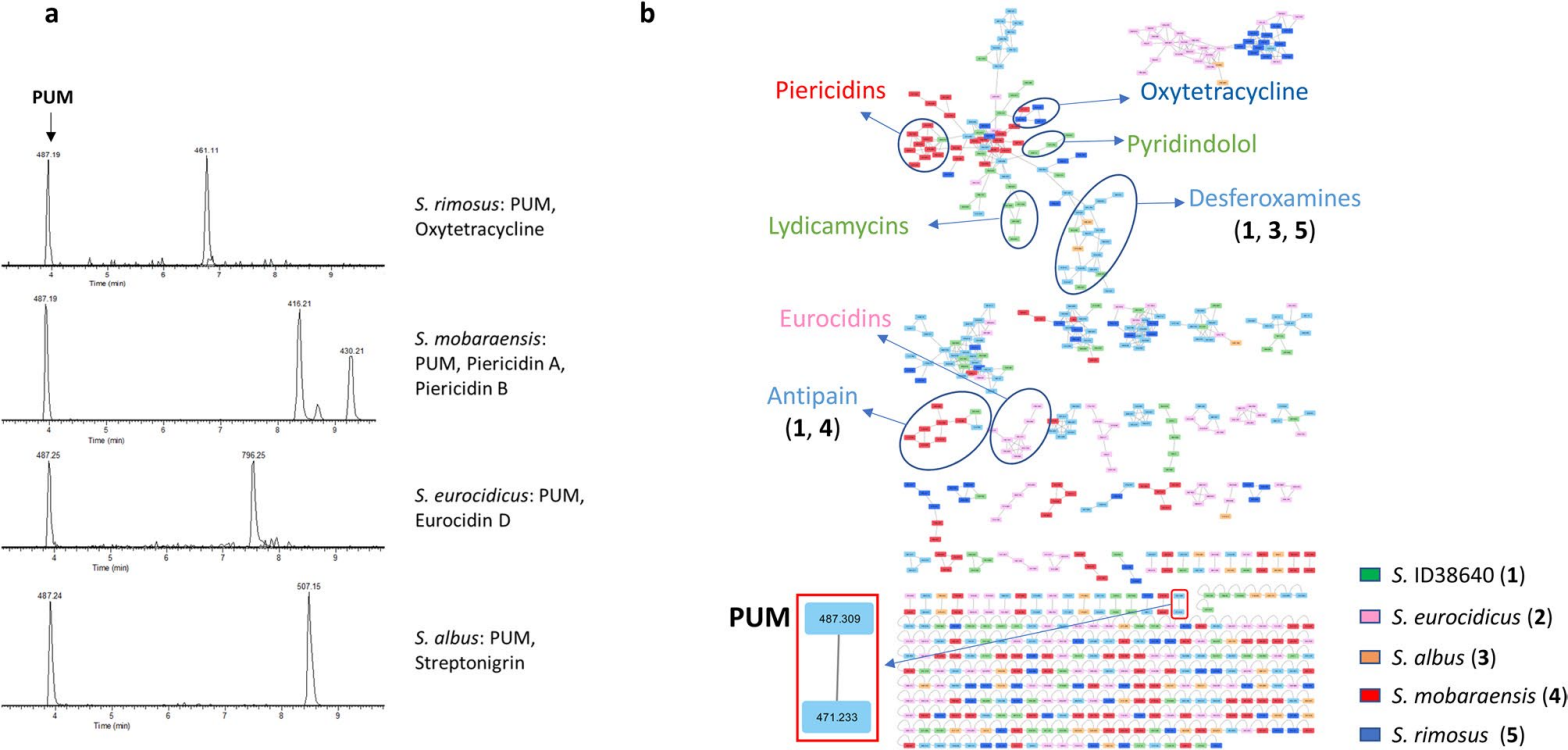
Analysis of PUM production in other *Streptomyces* strains. (**a**) Extracted ion chromatograms of PUM (*m/z* 487 [M + H]^+^) and strain specific metabolites from *S. rimosus, S. mobaraensis, S. eurocidicus* and *S. albus*. (**b**) Complete molecular network of two samples from each of the above PUM producers and from ID38640. Strain-specific features are color coded as: ID38640, green; *S. eurocidicus*, pink; *S. albus*, orange; *S. mobaraensis*, red; and *S. rimosus*, blue. Features detected in more than one strain are in light blue. All strains were cultivated in M8 medium and samples prepared at 72 h.

When grown in a single medium and analyzed at three different time points, each *Streptomyces* species, in addition to the expected metabolites oxytetracycline, piericidin, eurocidin D or streptonigrin, produced PUM (Fig. 4a), at a level comparable to *Streptomyces* sp. ID38640 (around 200 μM) for *S. rimosus* and *S. mobaraensis* or at approximately half these levels for *S. albus* and *S. eurocidicus*. These results indicated that the PUM BGC in these species is actively expressed and that PUM can be easily detected when properly looked for.

We were interested in establishing the phylogenetic relationship and the extent of shared metabolites of the five PUM producers. We thus applied autoMLST^37^ to construct a high-resolution species tree, which revealed ten major clades and three branches formed by a single strain each (Supplementary Fig. 8). *Streptomyces* sp. ID38640 belongs to clade 2 while, among the PUM producers reported above, only *S. rimosus* (clade 7) and *S. mobaraensis* (single-strain branch) were picked up. A phylogenetic tree of the five PUM producers showed that *Streptomyces* sp. ID38640 clustered with *S. rimosus*, while *S. eurocidicus* and *S. mobaraensis* formed a separate clade (Supplementary Fig. 9).

The ID38640 genome does not harbor BGCs for oxytetracycline, euricidin, piericidin or streptonigrin. In addition to PUM, only three additional BGCs are shared by the five strains: those for the frequently encountered *Streptomyces* metabolites geosmin and hopene; and that for the BGC labeled Other5 (Table 2). The latter BGC, consisting of a syntenic region of 8 conserved ORFs, is of unknown function and has been identified by antiSMASH as siderophore.

We next investigated whether the other PUM-producing strains shared other metabolites with *Streptomyces* sp. ID38640, using procedures similar to those described above. The resulting molecular network, represented in Fig. 4b, contains 630 features, including media components, of which 385 (61%) are organized in 62 molecular families. As highlighted by a red rectangle, a two-member family containing PUM and deoxy-PUM is found in samples from all strains. Of the families annotated in Fig. 1, lydicamycins, pyridindolol, ulleungdin and NK1 through NK3 remain ID38640-specific. Desferrioxamines were detected in *S. rimosus* and *S. albus*, while antipain was detected in *S. mobaraensis*. As described above, manual inspection of the LC–MS profiles showed ectoine in extracts from *S. rimosus* and *S. albus*. Additional metabolites were dereplicated in the samples and the corresponding BGCs were identified in the strain genomes (M.I., unpublished observations) but none of these additional molecules matched unannotated metabolites detected in *Streptomyces* sp. ID38640.

Taken together, the above results indicate that the PUM BGC is not restricted to a specific *Streptomyces* clade and that PUM is not regularly co-produced with other metabolites. It will be interesting to establish whether knockout in the PUM pathway in the other PUM producers can also alter their metabolic profiles.

## Conclusions

*Streptomyces* sp. ID38640 is a prolific and versatile producer of different metabolites, many of which could be detected only after selective blocks in the PUM pathway. While we do not yet understand why production of unrelated metabolites is significantly enhanced in different *pum* mutants, the approach used here might be a simple way of “catching two birds with a stone”, simultaneously elucidating a biosynthetic pathway of interest and observing alterations in the metabolome. Possible targets for this approach might include the other PUM producers reported in this study. In any case, the work presented here, along with our previous studies^38,39^, indicate that a metabolomic look at "old strains" can unveil previously overlooked chemistry, including novel metabolites. This sort of analyses will be undoubtedly facilitated by the growing Paired Omics Data Platform (https://pairedomicsdata.bioinformatics.nl)^40^.

## Materials and methods

### Bacterial strains and growth conditions

*Streptomyces* sp*.* ID38640, *S. flocculus* DSM 40313, *S. rimosus* ATCC 10970, *S. mobaraensis* DSM 40847, *S. eurocidicus* ATCC 27428 and the *pum* mutants were cultured as described^14^. Briefly, mycelium from BTT plates was inoculated in 50-mL Erlenmeyer flask containing 15 mL of seed medium (20 g/L dextrose monohydrate, 2 g/L yeast extract, 8 g/L soybean meal, 1 g/L NaCl, and 4 g/L CaCO_3_, pH 7.3), and incubated 72 h at 28 °C. The production media were M8^41^ and PumP1^14^, which were inoculated with a 10% volume of the seed culture.

### Construction of knockout mutants

The generation of *ΔpumF, ΔpumH* and *ΔpumL* strains followed described procedures^17^, which involved amplification of two ~1.0-kbp fragments (A and B) from genomic DNA using primers containing *Eco*RI and *Xba*I (fragment A) and *Xba*I and *Bam*HI (fragment B) tails (Supplementary Table 1), that were cloned into the *EcoRI–BamHI* sites of the vector pWHM3-*oriT-ΔXba*. In the resulting plasmid, the apramycin resistance gene was inserted at the *XbaI* site within the PCR-amplified *pum* segments to generate the knockout plasmid. The knockout plasmids were then introduced into *E. coli* ET12567/pUB307, whence they were conjugated into spores of *Streptomyces* sp. ID38640 as described^17^. Double-crossover mutants were identified through PCR with diagnostic primers.

### Genome sequence and bioinformatic analyses

Genome sequencing was performed by Cebitec Bielefeld University (Germany) using Illumina MiSeq/Genome Analyzer IIx/HiSeq 1000. BGCs were identified using the antiSMASH 5.0 at the default conditions^3^. BLAST analysis of individual CDSs was performed against the MIBiG database of known BGCs^21^ and against Protein Data Bank. Multilocus sequence analysis was performed with autoMLST in “denovo mode” and default settings^37^.

### Samples for LC–MS analysis

For PUM-related metabolite analysis, 0.5 mL of the culture was centrifuged at 13,200 rpm for 2 min and the supernatant was filtered through a 0.2-μm membrane (EuroClone), generating the SN sample. Full extracts (FE) were prepared by transferring a 0.5-mL sample from cultures into a 2-mL Eppendorf tube containing 0.5 mL MeOH. After 1 h at 55 °C under constant shaking, the sample was centrifuged for 10 min at 13,200 rpm and the supernatant was recovered and transferred into a 1.5-mL glass vial.

### Metabolite analysis

LC–MS analyses were performed with on a Dionex UltiMate 3000 coupled with an LCQ Fleet (Thermo scientific) mass spectrometer equipped with an electrospray interface (ESI) and a tridimensional ion trap. The column was an Atlantis T3 C18 5 mm × 4.6 mm × 50 mm maintained at 40 °C at a flow rate of 0.8 mL/min. Phases A and B were 0.05% trifluoroacetic acid in water and acetonitrile, respectively. SN samples were analyzed using the following gradient: 0 to 25% phase B in 4 min, followed by a 2-min wash at 90% and a 3-min re-equilibration at 0% phase B. The gradient used for FEs was a 14-min multistep program that consisted of 10, 10, 95, 95, 10 and 10% phase B at 0, 1, 7, 12, 12.5 and 14 min, respectively. UV–VIS signals (190–600 nm) were acquired using the diode array detector. The *m/z* ranges were set at 120–1500 and 200–2000 for SNs and FEs, respectively, with ESI conditions as follows: spray voltage of 3500 V, capillary temperature of 275 °C, sheath gas flow rate at 35 units and auxiliary gas flow rate at 15 units. High resolution mass spectra were acquired as described previously^42^.

### Metabolomic analysis

For the metabolomic analysis the Metabolomics-SNET-V2 (release_23) workflow was used. Parameters were adapted from the GNPS documentation: MS2 spectra were filtered so that all MS/MS fragment ions within ± 17 Da of the precursor *m/z* were removed. The MS/MS fragment ion tolerance and the precursor ion mass tolerance were set to 2.0 and 0.5 Da, respectively. Edges of the created molecular network were filtered to have a cosine score above 0.7 and at least 4 matched peaks between the connected nodes. The maximum size of molecular families in the network was set to 100. The MS2 spectra in the molecular network, filtered in the same manner as the input data, were searched against our internal library of 480 annotated metabolites. Reported matches between network and library spectra were required to have a score above 0.75 and at least 5 matching peaks. The molecular networks were visualized using Cytoscape.

### Metabolite quantification

Pseudouridine-containing intermediate were quantified by HPLC assuming an identical chromophore as PUM, against a purified pseudouridimycin internal standard. Relative amounts of the other metabolites were estimated as peak intensity ratio to those observed in WT strain.

### Nucleotide sequence accession number and Paired Omics Data Platform project identifier

The genome sequence has been deposited in GenBank under the accession CP049782 as BioProject PRJNA609626. A subset of metabolomic data has been deposited in the Paired Omics Data Platform (Metabolomics project identifier c86fdc82-0d18-45d0-aa30-1f877c1cd3fc.2).

## Supplementary information

Supplementary information.
