# Supplementary material for: Blocks in the pseudouridimycin pathway unlock hidden metabolites in the Streptomyces producer strain
Source: Sci Rep. 2021 Mar 12;11:5827. doi: 10.1038/s41598-021-84833-2 (PMC7955054; doi:10.1038/s41598-021-84833-2)
Supplement: Supplementary file 1 — Supplementary information. [file 41598_2021_84833_MOESM1_ESM.docx]

**Supplementary Information for**

**Blocks in the pseudouridimycin pathway unlock hidden metabolites in the Streptomyces producer strain**

Marianna Iorio^a^, Sahar Davatgarbenam^a^, Stefania Serina^a^, Paolo Criscenzo^a^, Mitja M. Zdouc^a,b^, Matteo Simone^a^, Sonia I. Maffioli^a^, Richard H. Ebright^c^, Stefano Donadio^a^ and Margherita Sosio^a*^

^a^ NAICONS, viale Ortles 22/4, 20139 Milano, Italy; ^b^ Swammerdam Institute for Life Sciences, University of Amsterdam, Science Park 904, 1098 XH, Amsterdam, The Netherlands; ^c^ Department of Chemistry and Waksman Institute, Rutgers University, Piscataway, NJ 08854, USA.

* corresponding author: [msosio@naicons.com](mailto:msosio@naicons.com)


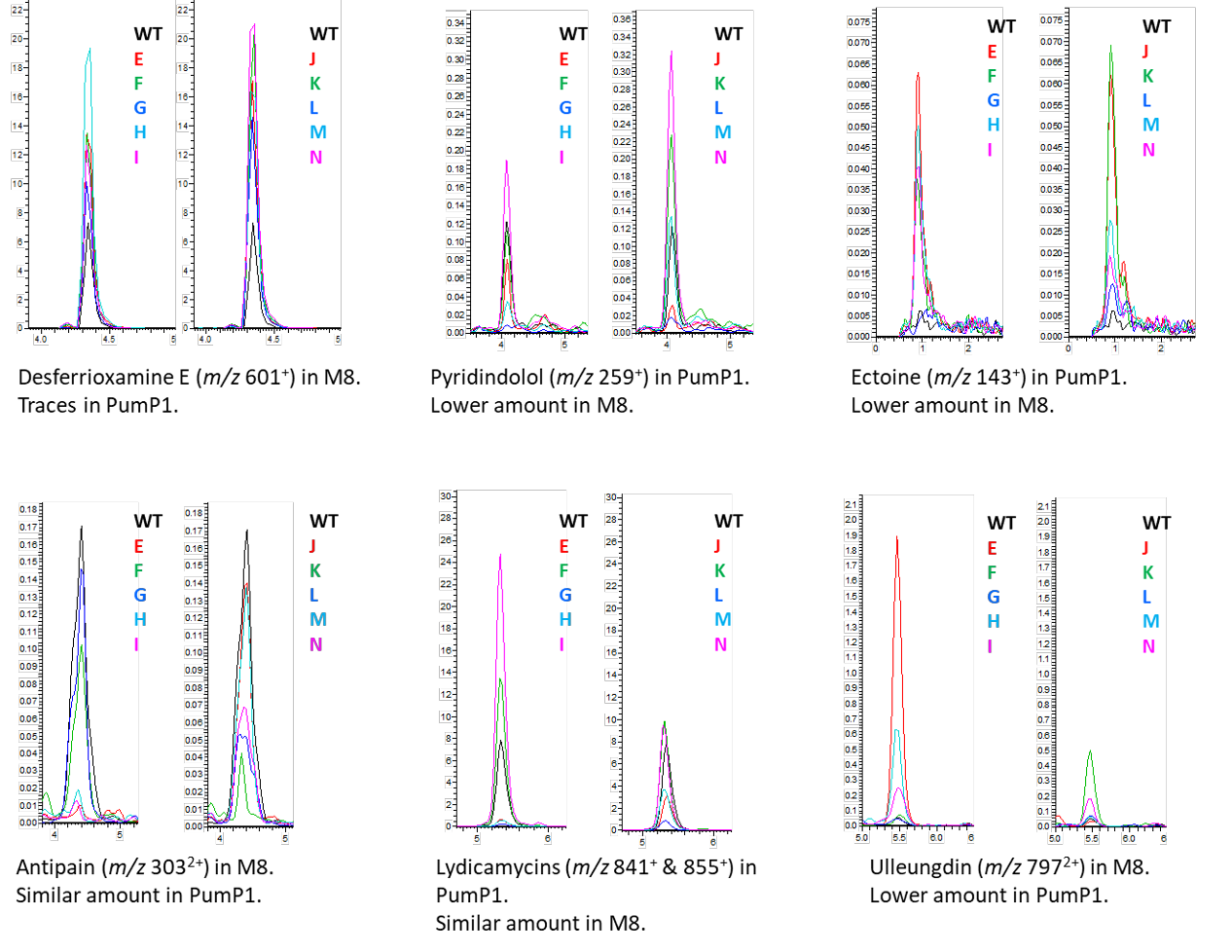


**Supplementary Figure 1**. Comparison of extracted ion chromatograms related to desferrioxamine E(a), pyridindolol (b), ectoine (c), antipain (d), lydicamycin (e) and ulleungdin (f) in the best medium from all the mutants and the wild type strain. Each comparison is split into two panels for clarity, with the WT added for correlation. *pum* mutants are designated by their letter suffix and color coded.


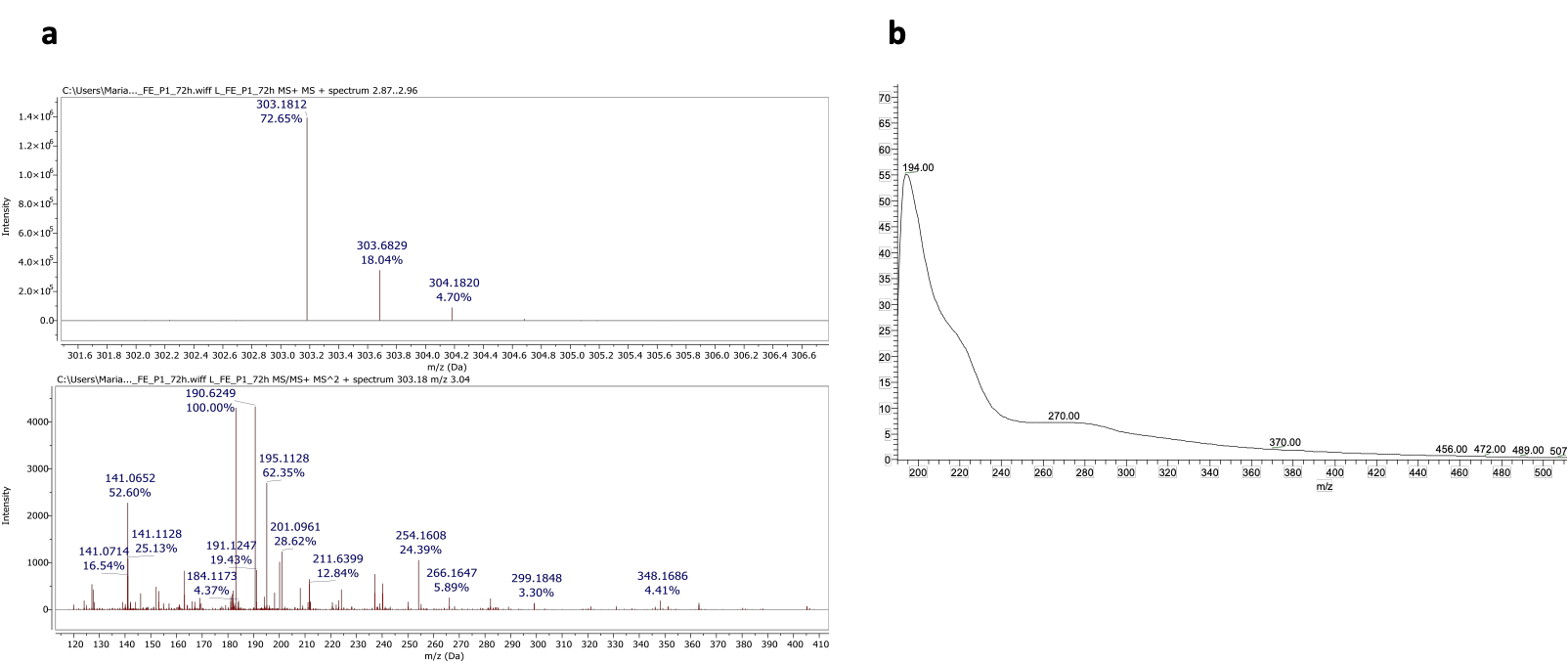


**Supplementary Figure 2.** HR-MS and fragmentation spectrum of antipain (a) and its UV-Vis spectrum (b).


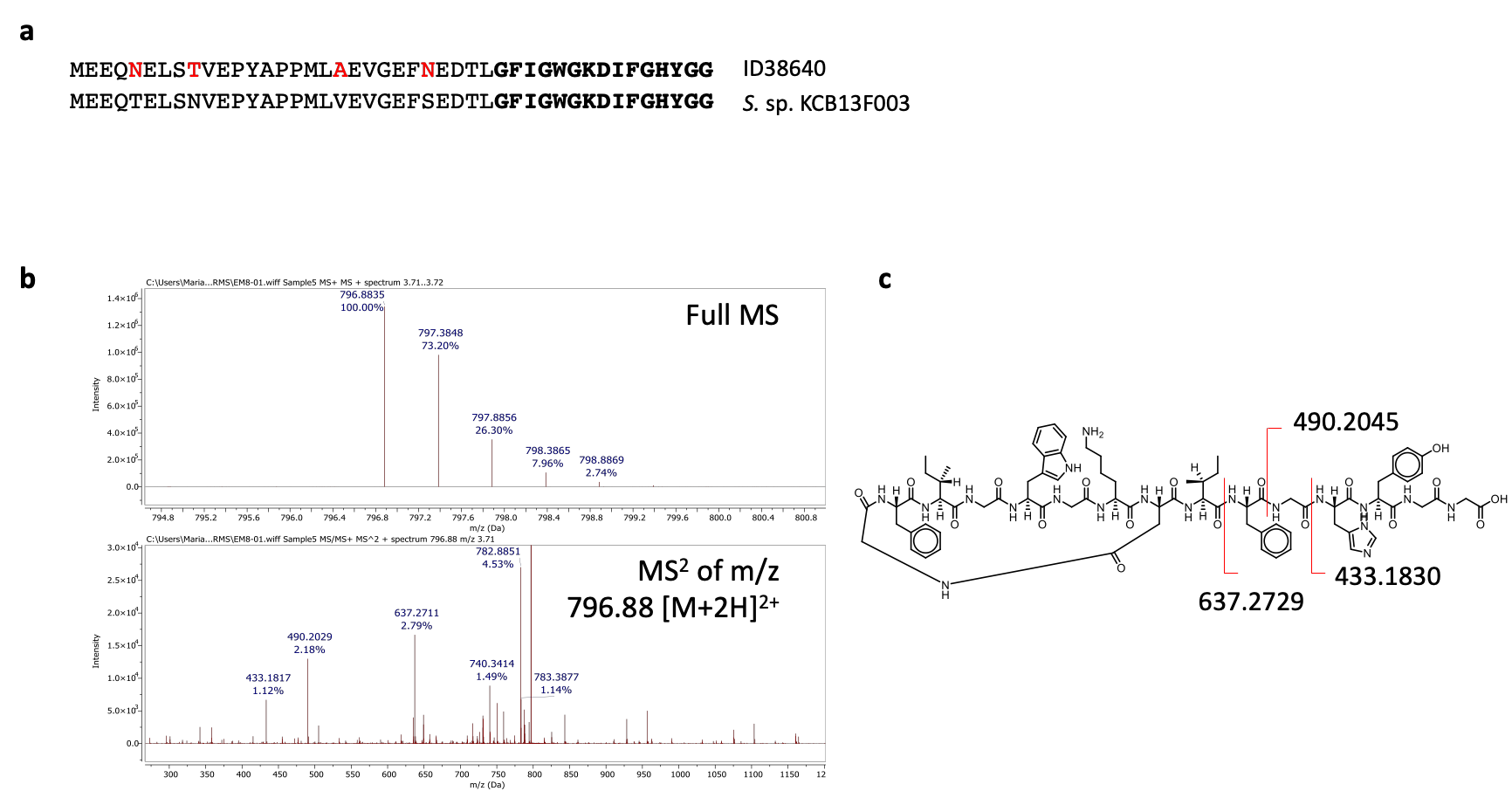


**Supplementary Figure 3.** a) Precursor peptide of ulleungdin in ID38640 and in *Streptomyces* sp. KCB13F003. The different amino acids residues between the two leader sequences are highlighted in red type, while the core peptide is in bold type. b) HR-MS and fragmentation spectrum of ulleungdin and (c) its annotated fragmentation pathway.


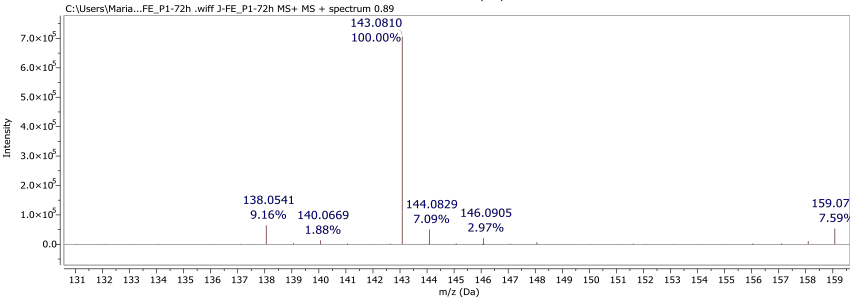


**Supplementary Figure 4.** HR-MS of ectoine.


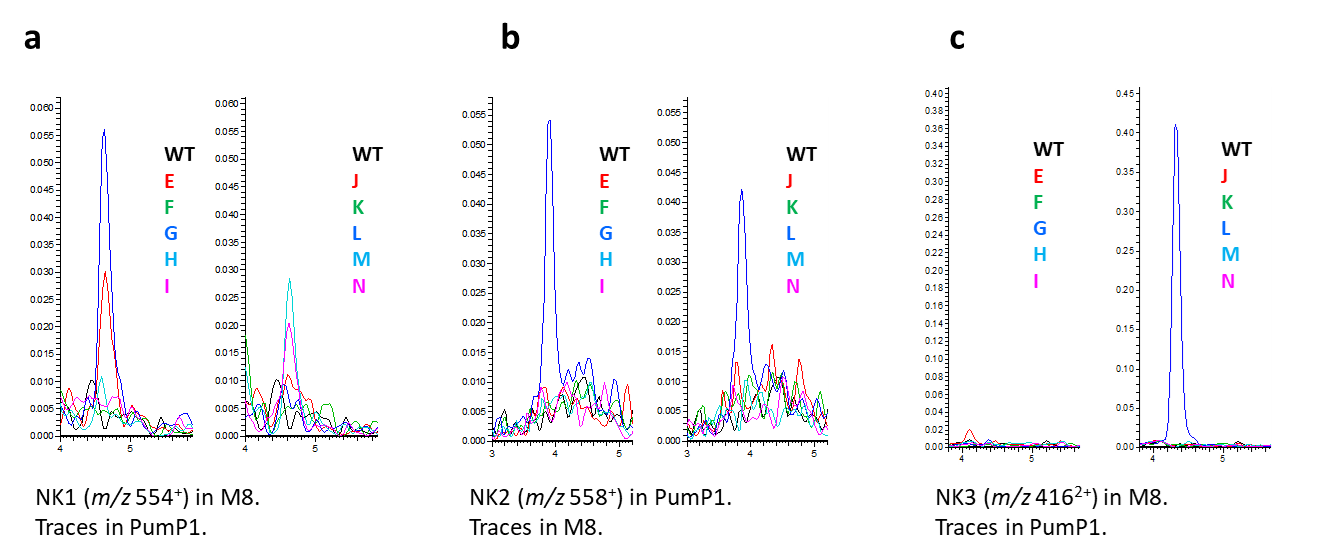


**Supplementary Figure 5.** Comparison of extracted ion chromatograms related to the most representative species of the unannotated molecular families NK1 (a), NK2 (b) and NK3 (c) in the best medium from all the mutants and the wild type strain. Mutants labeled as in Supplementary Fig. 1.


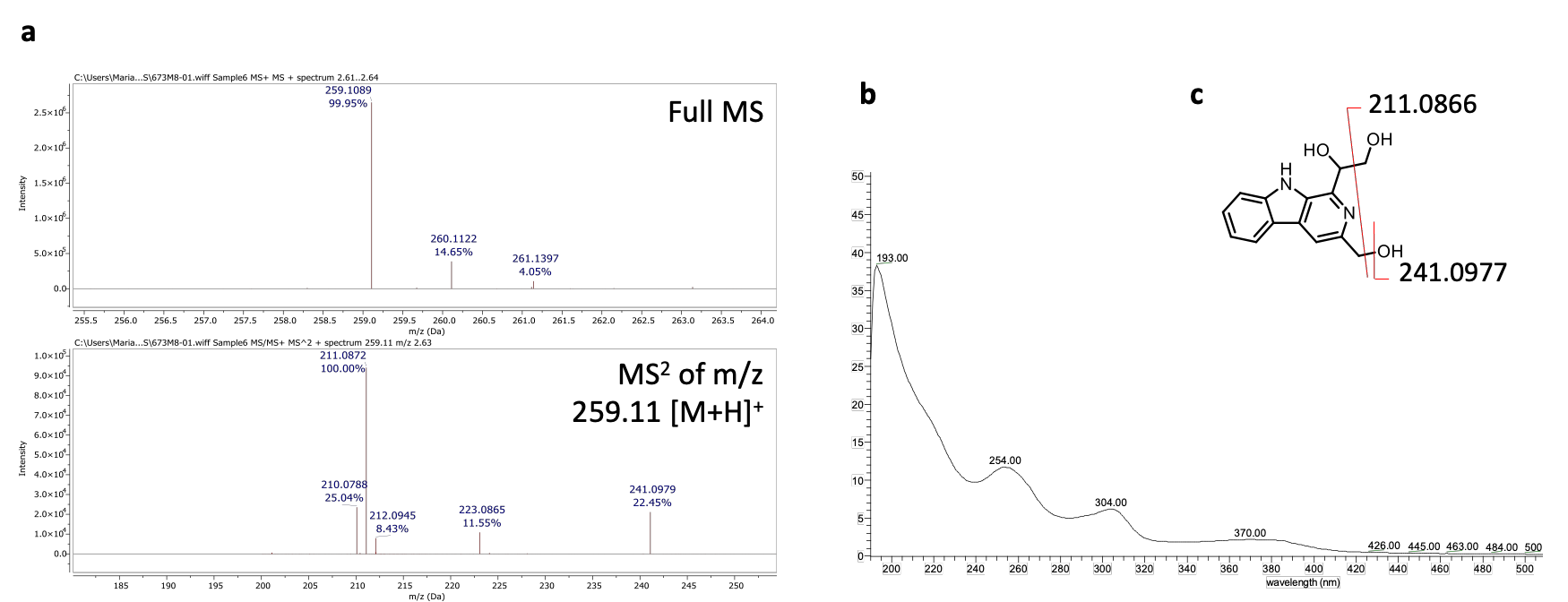


**Supplementary Figure 6.** a) HR-MS and fragmentation spectrum of pyridindolol, its UV-Vis spectrum (b) and annotated fragmentation pathway (c).


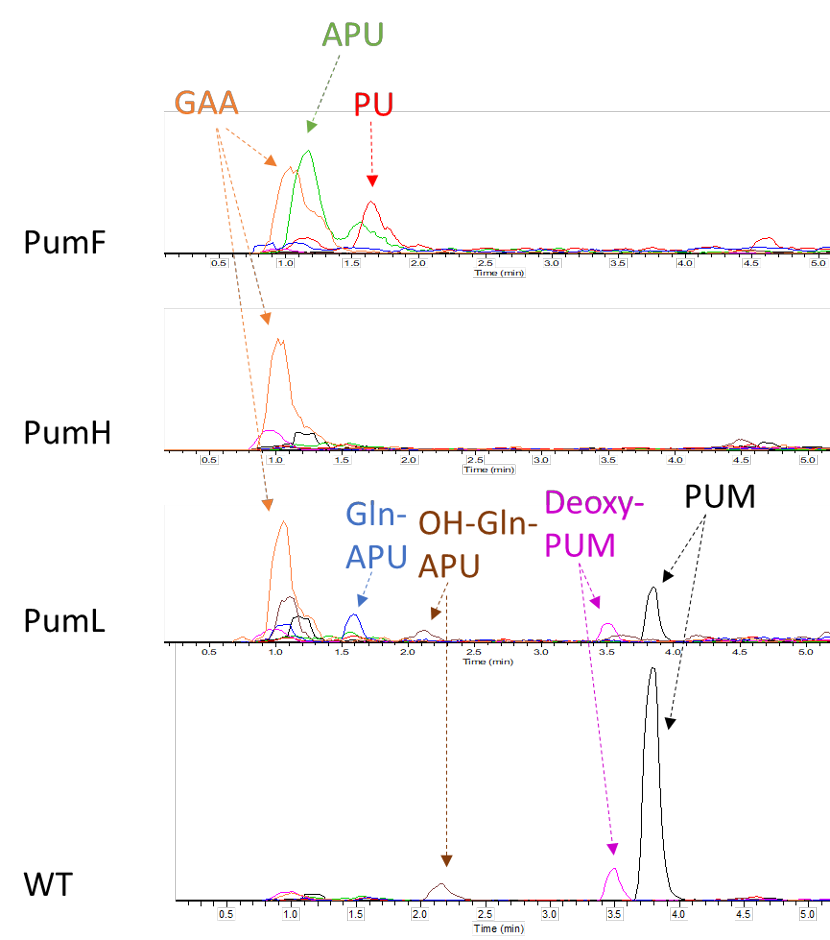


**Supplementary Figure 7.** Extracted ion chromatograms of the Δ*pumF*, Δ*pumH* and Δ*pumL* mutants. The analyses show pseudouridimycin (PUM, *m/z* 487 [M+H]^+^, black line), pseudouridine (PU, *m/z* 245 [M+H]^+^, red line), aminopseudouridine (APU, *m/z* 244 [M+H]^+^, green line), Gln-APU (*m/z* 372 [M+H]^+^, blue line), OH-Gln-APU (*m/z* 388 [M+H]^+^, brown line), deoxy-PUM (*m/z* 471 [M+H]^+^, pink line) and guanidinoacetic acid (GAA, *m/z* 344 [M+2TFA-H]^-^, orange line).


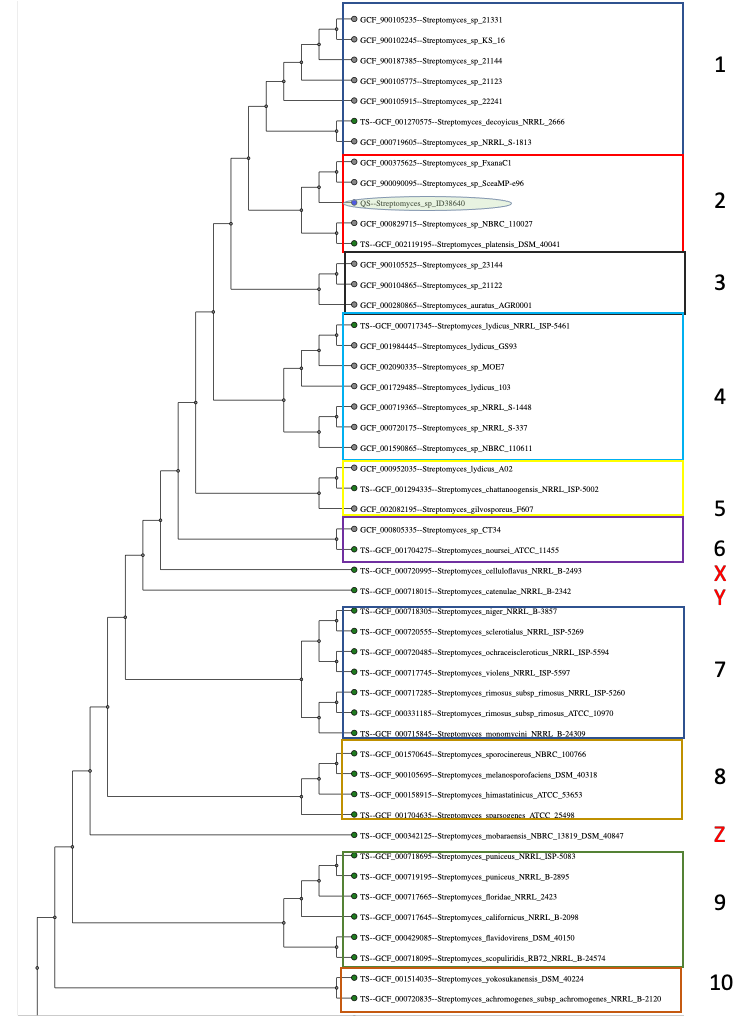


**Supplementary Figure 8.** Maximum likelihood tree of 50 members of the genus *Streptomyces* based on 100 concatenated housekeeping genes identified by autoMLST. Color code is representing the nine major clades while X, Y and Z designated the three single strain branches. ID38640 is highlighted by a green circle.


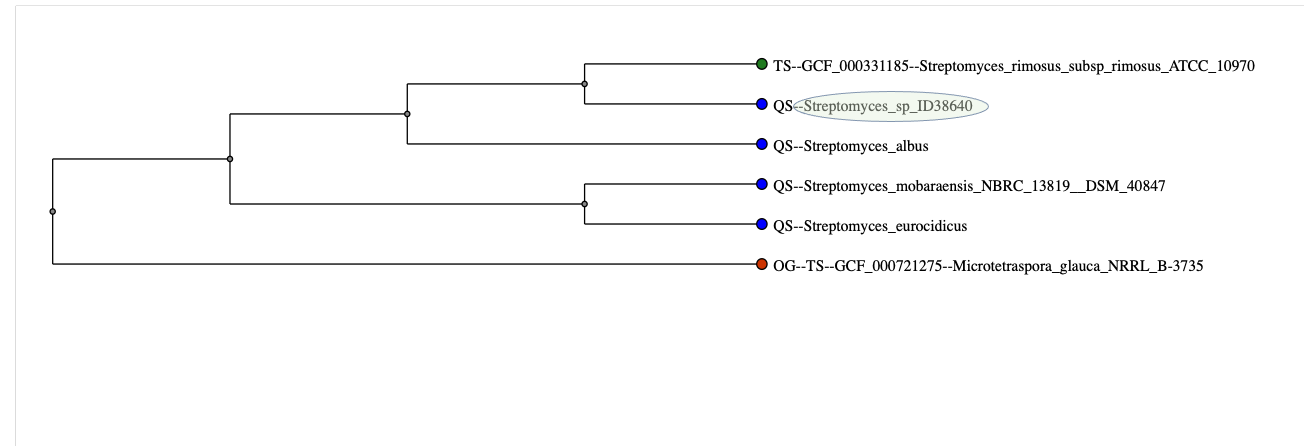


**Supplementary Figure 9.** Maximum likelihood tree generated of ID38640, *S. eurocidicus,* *S. albus*, *S. mobaraensis*, and *S. rimosus* (*Microtetraspora glauca* NRRL B-3735 used as the outgroup), based on 100 concatenated housekeeping genes identified by autoMLST. ID38640 is highlighted by a green circle.


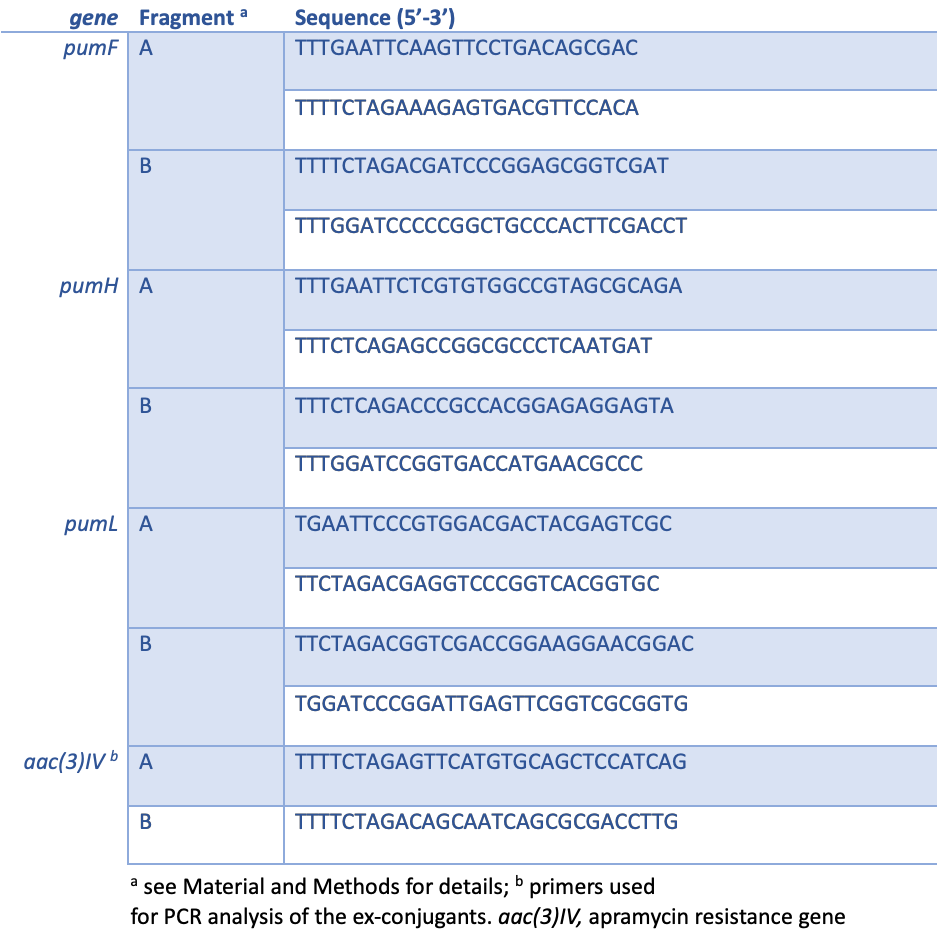


**Supplementary Table 1.** List of primers used for knock outs experiments.
